# Exploring Reaction Conditions to Improve the Magnetic Response of Cobalt-Doped Ferrite Nanoparticles

**DOI:** 10.3390/nano8020063

**Published:** 2018-01-25

**Authors:** Itziar Galarreta, Maite Insausti, Izaskun Gil de Muro, Idoia Ruiz de Larramendi, Luis Lezama

**Affiliations:** 1 Department of Inorganic Chemistry, University of the Basque Country, UPV/EHU, Bº Sarriena, 48970 Leioa, Spain; itziar.galarreta@ehu.eus (I.G.); izaskun.gildemuro@ehu.eus (I.G.d.M.); idoia.ruizdelarramendi@ehu.eus (I.R.d.L.); luis.lezama@ehu.es (L.L.); 2 BCMaterials, Basque Center for Materials, Applications & Nanostructures, UPV/EHU Science Park, Bº Sarriena, 48970 Leioa, Spain

**Keywords:** Co-doped ferrite, magnetic nanoparticles, superparamagnetism, anisotropy energy, magnetic properties

## Abstract

With the aim of studying the influence of synthesis parameters in structural and magnetic properties of cobalt-doped magnetite nanoparticles, Fe_3−*x*_Co*_x_*O_4_ (0 < *x* < 0.15) samples were synthetized by thermal decomposition method at different reaction times (30–120 min). The Co ferrite nanoparticles are monodisperse with diameters between 6 and 11 nm and morphologies depending on reaction times, varying from spheric, cuboctahedral, to cubic. Chemical analysis and X-ray diffraction were used to confirm the composition, high crystallinity, and pure-phase structure. The investigation of the magnetic properties, both magnetization and electronic magnetic resonance, has led the conditions to improve the magnetic response of doped nanoparticles. Magnetization values of 86 emu·g^−1^ at room temperature (R.T.) have been obtained for the sample with the highest Co content and the highest reflux time. Magnetic characterization also displays a dependence of the magnetic anisotropy constant with the varying cobalt content.

## 1. Introduction

Nanosized magnetite structures are one subject of interest in biomedicine, as many diseases can actually be diagnosed and treated by magnetic nanoparticle (NP)-based therapies, which include magnetic resonance imaging [1], drug targeting [2,3], magnetic separation [4], or magnetic hyperthermia [5]. In this last case, superparamagnetic nanoparticles are able to induce heating under the application of an alternating magnetic field, and so magnetic nanoparticles can be applied as therapeutic agents in some cancer treatments. The values of temperature rise are closely related to the characteristics of the sample (nature and homogeneity of the nanoparticles, composition, magnetocrystalline anisotropy, saturation magnetization, average particle size, shape, or size dispersion) and also to the excitation conditions of the alternating magnetic field used (amplitude, *H*, and frequency, *ν*) [6,7].

Magnetite nanoparticles, Fe_3_O_4_, due to their biocompatibility, stability, and low cytotoxicity, together with high-saturation magnetization, high remanence, and moderate anisotropy constants are the most promising ones for biomedical applications. Additionally, their magnetic properties can be tuned by partially replacing the divalent or trivalent iron ions with cations like Zn^2+^, Mn^2+^, Co^2+^, and Ni^2+^ [8,9]. In this sense, cobalt ferrite can be considered an interesting material as the Co^2+^ cation is more anisotropic and doped ferrites would allow increasing the magneto-crystalline anisotropy maintaining similar magnetic moments. Nevertheless, the different occupation of Co^2+^ ions in the tetrahedral (A), and octahedral positions (B), in ferrites (general formula AB_2_O_4_), would determine the magnetization in the ferrimagnetic structure according to the Yafet-Kittle-like model [10]. It has been found that cobalt ferrites present a partially-inverted spinel structure, where bivalent cations are present both in the tetrahedral (A) and octahedral positions (B), [Fe^3+^Co^2+^*_y_*]_A_[Fe^2+^_1−*x*−*y*_Co^2+^*_x_*,Fe^3+^]_B_O_4_. Although cobalt(II) ions exhibit preference for the octahedral sites, this distribution can be altered depending on the synthesis conditions [11].

In recent years, researchers have presented a significant interest in preparing CoFe_2_O_4_ nanostructures with different sizes, morphology, and cobalt stoichiometry for potential application as biomedical materials [12]. Among other methods, thermal decomposition of iron(III) acetylacetonate, Fe(acac)_3_, and cobalt(II) acetylacetonate, Co(acac)_2_, or metal oleates in high-temperature solvents have been used for preparing CoFe_2_O_4_ of different shapes and sizes by changing reaction times, temperature, surfactant concentration, solvent, or precursor ratios [12,13]. This method usually yields more homogeneous nanoparticles, although it requires an ulterior transference to water media. For CoFe_2_O_4_ nanoparticles with sizes of 4 nm, specific absorption rate (SAR) values of 35.24 W/g at a fixed frequency of 276 kHz and at 419 Oe and appreciable cell viability for in vivo applications have been observed [8].

The influence of Co doping in the magnetic behavior of ferrite nanostructures has also been a topic of investigation. In this way, Co-doped maghemite nanoparticles (Co*_x_*Fe_(8/3−2*x*/3)_O_4_) of 5 nm have been prepared in the range 0 < *x* < 1 maintaining constant the structural and morphological parameters. The large values of magnetization obtained were explained because of the different occupation of tetrahedral and octahedral sites in the structure [14]. Cube-shaped Co*_x_*Fe_3−*x*_O_4_ nanocrystals (0.1 < *x* < 0.7) of varying size have also been synthesized tuning the heating ramp or the solvent nature. They seem very promising materials for magnetic hyperthermia as they exhibit SAR values of 915 W·g^−1^ at 105 kHz and 32 kA·m^−1^, which can be ascribed to the core-shell structure, the high coercitivity and magnetization, and the peculiar shape of the samples [15]. Nevertheless, the real composition of the samples in Co together with the influence of the morphology and the dependence of both factors on magnetization, has not been explored in detail.

To attain a better understanding of Co content and the reaction conditions in the structural and magnetic properties of Co-substituted ferrites, we have prepared Co*_x_*Fe_3−*x*_O_4_ nanoparticles by thermal decomposition of Fe(acac)_3_ and Co(acac)_2_ in benzyl ether at different reflux times, from 30 to 120 min. Taking into account the effect of synthetic parameters on the magnetic response, and reaction times of 60 min have been selected in order to analyze the influence of cobalt content in the ferrite lattice over the magnetization values. The samples so obtained have been named as Co*_x_*_*t*, where *x* is the theoretical stoichiometry of Co in the formula and t corresponds to the minutes of reflux at high temperatures. The purity, structure, and phases of synthetized materials were analyzed using X-ray diffraction (XRD). Size and morphology were studied by transmission electron microscopy (TEM). The organic component of the samples was quantified by thermogravimetric analysis, the saturation magnetization of samples were obtained using a vibrating sample magnetometer (VSM). In addition, a rigorous magnetic study of the samples was performed by means of a superconducting quantum interference device (SQUID) magnetometer and an electron magnetic resonance (EMR).

## 2. Results

### 2.1. Structural and Chemical Characterization

The thermal decomposition of the metallo-organic precursors yields Co*_x_*_*t* ferrite nanoparticles surrounded by oleic acid (*x* = 0.01, 0.04, 0.1, 0.15; *t* = 30, 45, 60, 75, 90, 105, 120 min). Inductively-coupled plasma-atomic emission spectroscopy (ICP-AES) was firstly used to analyze the synthesized oxides (Table 1). Even though cobalt doping has been corroborated, nanoparticles show a cobalt-deficient composition. Nanoparticles with nominal composition *x* = 0.15 present cobalt contents in the 0.08–0.16 range. A deficiency of cobalt has been previously established in similar ferrite nanoparticles prepared with oleic acid (OA), oleylamine (OLA), and 1,2-hexadecanediol (HDD) as surfactants [16]. In fact, Crouse et al. determined that modifying different synthesis parameters, such as the co-surfactant or the proportion of metallic precursors, the incorporation of cobalt cations into the ferrite lattice could be modified, although a steady reduction in Co content in all analyzed compositions was observed [17]. This study also shows that low concentration of HDD yields smaller nanoparticles, however, particle size distribution is affected. On the other hand, Shemer et al. noticed the effect of the diol chain length on the composition of cobalt-doped ferrites [18]. They reported that the presence of HDD significantly reduces the amount of Co^2+^ ions in the ferrite, determining that the presence of diol-type co-surfactants is directly related with the growth mechanism of the nanoparticles. In fact, the incorporation of cobalt in the ferrite structure could be related to the decomposition temperature of the metal precursors and, in the case of Co(acac)_2_, is higher than for Fe(acac)_3_ [13,19]. Hence, the use of a high-temperature boiling point solvent and an increase in the reflux time would give rise to a greater incorporation of Co. Nevertheless, analysis results do not show this trend with the reflux time, probably due to the influence of the solvent.

**Figure 1:**
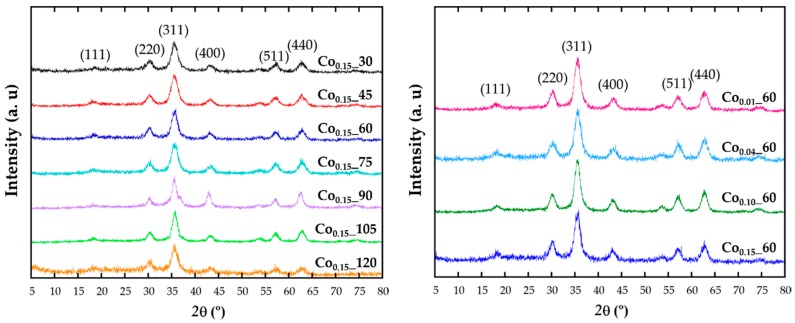
X-ray diffraction pattern of the samples obtained with different reflux times (**left**) and at varying the cobalt content (**right**).

X-ray diffraction (XRD) patterns have been recorded in order to identify the structure (Figure 1). The intensity and peak positions of the diffraction patterns are in good agreement with the face-centered cubic phase of magnetite (Fd-3m, JCPDS No. 89-0691). All the major diffraction peaks have been indexed with (111), (220), (311), (222), (400), (422), (511), and (440) crystallographic planes and no reflections corresponding to other secondary phases appear. The effect of cobalt doping cannot be distinguished in the patterns, being the observed broad diffraction maxima of those expected for such small crystalline domains.

Diffraction data have been Rietveld [20] fitted using the FullProf program [21] and the obtained refined cell parameters are included in Table 1. Taking into account that the ionic radius of Co^2+^ (0.745 Å) is slightly smaller than for Fe^2+^ (0.78Å), it could be expected that the cell parameters of a solid solution with increasing cobalt content would follow Vegard’s law [22]. Thus, by doping the ferrite lattice with Co, unit cell parameters should slightly reduce comparing with those from Fe_3_O_4_ structure (Fd-3m; a = 8.387 Å; JCPDS No. 89-0691). It can be observed that although no direct relation with the theoretical nominal composition can be deduced, by fitting the diffraction data a slight and general reduction of cell parameters, compared with those of magnetite, is observed for all doped samples, corroborating the inclusion of cobalt in the unit cell.

This fact is indicative of the presence of cobalt in the spinel structure, being the key factor for a proper understanding of the effect of doping on the magnetic response, as magnetic properties directly depend on the cation distribution in octahedral or tetrahedral positions of this spinel structure. In the synthesis process of Co*_x_*_*t* samples, a Co(II) precursor has been used in a rather reductive environment, so the presence of only Co^2+^ cations is expected. According to crystal field theory, Co^2+^ cations would occupy octahedral positions [11], although it has also been reported that when Co(III) ions coexist with Co(II) in the spinel structure, the Co^3+^ cations exclusively occupy octahedral sites, displacing Co^2+^ cations to tetrahedral positions [23,24]. Taking into account the above considerations, and that in previous studies of doped ferrite nanoparticles for low cobalt concentrations Co^2+^ cations would rather occupy the octahedral sites [25], a priori we consider the same assumption for Co*_x_*_*t* nanoparticles.

**Figure 2:**
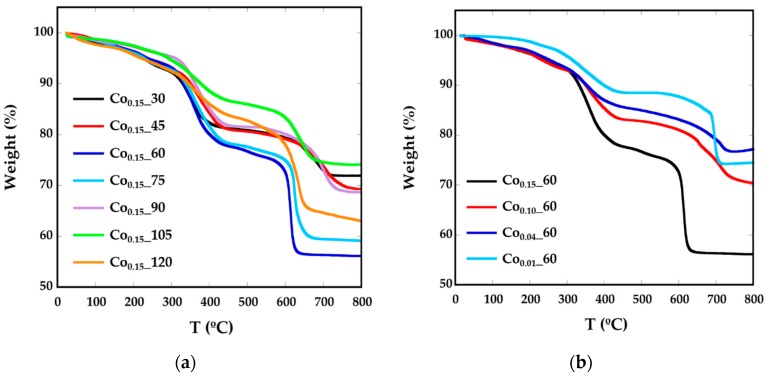
TGA curves of Co*_x_*_*t* phases for different (**a**) reflux times and (**b**) Co concentrations.

As previously mentioned, nanoparticles have been prepared in the presence of surfactants, so they are surrounded mainly by oleic acid. In order to calculate the amount of organic matter around the particles, thermogravimetric analysis (TGA) under Ar flow has been performed (Figure 2, Table 1). The decomposition curves for all the samples present three different steps. Up to 200 °C, the mass loss would correspond to degradation of the residual solvents and adsorbed atmospheric water. In the interval between 300 °C and 400 °C decomposition of the weakly attached surface functional coating or capping molecules (oleic acid molecules and oleylamine) occurs, as boiling temperatures of oleic acid (OA) and oleylamine (OLA) are 360 °C and 364 °C, respectively. The mass loss of about 20% above 600 °C can be attached to the decomposition of tightly-linked molecules or intermediate carbonates, together with a process of evolution of the ferrite inorganic core [26,27]. To confirm this fact, the residue obtained at TGA has been characterized by XRD (Supplementary Information, Figure S1) and in the diffraction pattern, together with the presence of the Fe_3_O_4_ phase, there is a significant concentration of FeO (JCPDS 75-155) and CoFe (JCPDS 44-1433), which is in good accord with the evolution of the inorganic Co*_x_*Fe_3−*x*_O_4_ core into reduced phases.

### 2.2. Morphological Characterization

**Figure 3:**
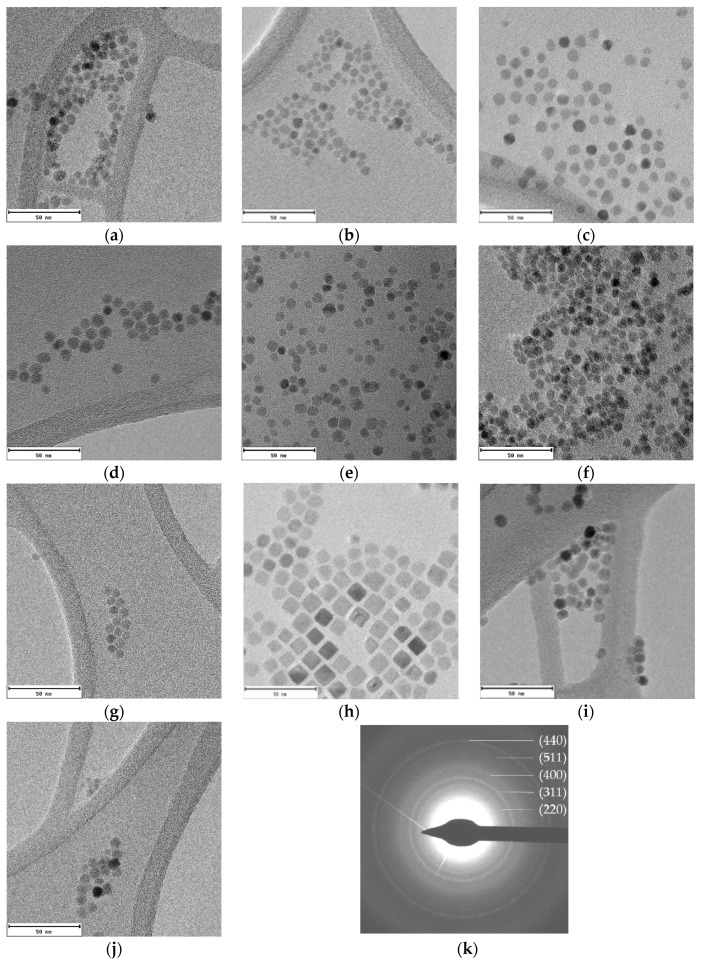
TEM images of cobalt-doped ferrite nanoparticles synthesized using different reflux times: (**a**) Co_0.15__30; (**b**) Co_0.15__45; (**c**) Co_0.15__60; (**d**) Co_0.10__60; (**e**) Co_0.04__60; (**f**) Co_0.01__60; (**g**) Co_0.15__75; (**h**) Co_0.15__90; (**i**) Co_0.15__105 and (**j**) Co_0.15__120; and (**k**) the electron diffraction pattern obtained for the Co30 sample is also provided. Scale bar: 50 nm.

TEM micrographs of all samples show well-dispersed homogeneous nanoparticles (Figure 3). The lack of aggregates suggest that cobalt-doped ferrite nanoparticles are well capped by the organic molecules. The average diameters obtained from TEM image analysis vary from 6 to 11 nm (Table 1). Particle size distributions have been included in Supplementary Information (Figure S2).

The electron diffraction pattern for the Co_0.15__30 sample appears in Figure 3k. The calculated d-spacings match well with (220), (311), (400), (511), and (440) planes corresponding to magnetite (Fe_3_O_4_) [28].

As can be noticed in Figure 3, nanoparticles with different shape and size are obtained for changing reaction times. Although a direct relation between reflux times and sizes is not observed, in good agreement with data in the literature [16,29], the shape of the nanoparticles certainly depends on that time. At low reaction times spherical nanoparticles appear and, at increasing reaction times, nanocubes and, by prolonging the reaction time, spheric/cuboctahedral nanoparticles appear. This evolution has also been reported by other authors [12,30]. In fact, these changes in the morphology have been related to both the benzyl ether solvent amount and the refluxing time. Firstly, small spherical nanoparticles are formed and can evolve into cubes, and even stars, as the reaction time is increased. Another essential factor is the relative surfactant concentration, since it has been observed that, at low concentrations of OA and OLA, the main morphology is spherical, but increasing the surfactant concentration nanocubes are obtained due to the predilection of the carboxylic group of OA to link to the {100} plane, hampering the growth in that direction [31].

However, the –NH_2_ group in oleylamine binds weakly and isotropically onto the surface of particles. In fact, Sun et al. reported the synthesis of nanocubes with OA surfactant to metal precursor ratios greater than 3:1 [32]. In this work, the OA/metal precursor relationship has remained at 2, so spherical nanoparticles were expected.

### 2.3. Magnetic Characterization

**Figure 4:**
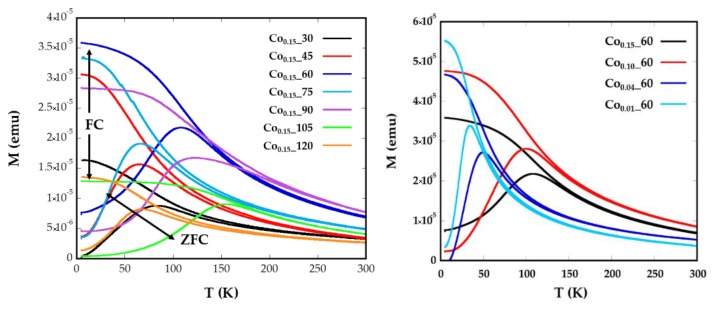
Temperature dependence of *ZFC* and *FC* magnetizations for samples Co*_x_*_*t* for different (**a**) reflux times and (**b**) Co concentrations, measured at a magnetic field of 10 Oe.

Magnetization versus temperature after zero field (*ZFC*) and field cooling (*FC*) (Figure 4) were obtained under a constant magnetic field of 10 Oe from well-dispersed and dried sample over filter paper, in order to avoid polar interactions between nanoparticles. In a system with a set of nanoparticles of identical size, the maximum of the *ZFC* curve is assumed to be equal to the blocking temperature of sample, *T_B_*, which is associated with the blocking-unblocking process of the nanoparticles magnetic moments depending on the thermal energy. According to Stoner–Wohlfarth theory [33], blocking temperatures are proportional to the energy barrier *E_B_* between equivalent easy directions:

(1) $$T_{B}=\frac{E_{B}}{\ln(\tau_{m}/\tau_{0})k_{B}}$$

where *k_B_* is the Boltzmann constant and *τ_m_* represents the inverse of the frequency of jump attempts, assuming an Arrhenius-type time relaxation where *τ*_0_ is the time window of the experiment. The effective magnetic anisotropy, *K_eff_*, is defined by *E_B_* = *K_eff_V*, being *V* the volume of the particle and it is determined by the shape, surface and magneto-crystalline anisotropy (*K_c_*) of the material [34]. Nevertheless, another contribution to the effective magnetic anisotropy due to dipolar interactions among the nanoparticles can appear. It is usually assumed in Equation (1) that *τ_m_* = 100 s and *τ*_0_ = 10^−9^ s (Supplementary Information, Model S1).

In Figure 4a broad maxima of *ZFC* curves can be observed for Co_0.15__60, Co_0.15__90, and Co_0.15__105 nanoparticles, which are shifted to higher temperatures than the maxima of the other samples. The increase of *T_B_* could also be related to the higher particle sizes (8(1), 11(1), and 8(1) nm) exhibited by samples Co_0.15__60, Co_0.15__90, and Co_0.15__105, respectively [35]. These temperatures assigned to the maximum of the *ZFC* curves, *T_B_*, are slightly displaced from the temperatures where *ZFC* and *FC* split, *T_irrv_*, which are indicative of the deblocking of the magnetic moment of the largest nanoparticles. In the Co_0.15__90 sample the difference observed in the *ZFC*-*FC* curve between *T_B_* = 123 K and *T_irrv_* = 220 K (Figure 4) is indicative of a broad particle size distribution and, consequently, a distribution of the anisotropy energy barriers. Thus, in order to properly determine and compare *T_B_* values of the samples, it is helpful to calculate the mean blocking temperatures <*T_B_*>. Additionally, the distribution of the energy barriers has been obtained by a direct subtraction of the field cooled and zero field cooled branches of magnetization versus temperature measurements through the derivative −*d*(*χ_FC_*-*χ_ZFC_*)/*dT* curves [36,37] (Supplementary Information, Model S1). This difference is proportional to the number of particles in the blocked regime that decreases when the thermal energy overcomes the anisotropy energy of each nanoparticle at increasing temperatures. From these calculations, average blocking temperatures <*T_B_*> have been obtained for all the samples (Table 2, Supplementary Information, Figure S3). These <*T_B_*> values can be used to estimate the effective anisotropy constants by means of Equation (1), however, from this equation the calculated *K_eff_* would be indicative of the anisotropy at temperatures close to the maximum of the *ZFC*, which varies from one sample to another. These data are not comparable, as Co containing nanoparticles show a significant variation of anisotropy constant with temperature [34]. Therefore, the determination of effective anisotropy constants at 0 K for all samples would become more useful in order to establish comparisons among them.

**Figure 5:**
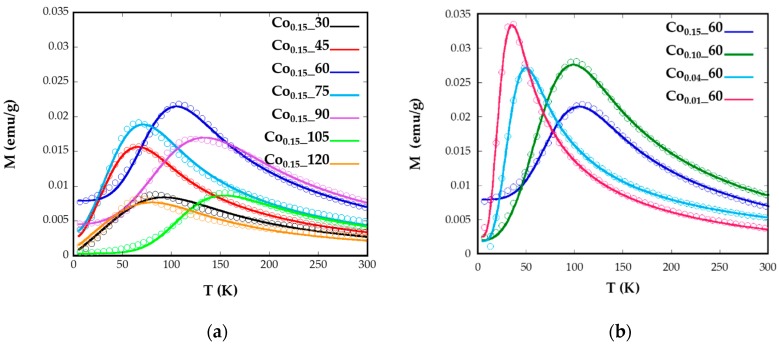
Experimental *ZFC* measurements (circular markers) with the fits (solid lines) using Equation (2) for samples Co*_x_*_*t* for different (**a**) reflux times and (**b**) Co concentrations.

For this purpose, the *ZFC* curve has been fitted with the following equation (Equation (2)) composed of one term corresponding to the thermally-activated NPs and another term to the blocked NPs (Supplementary Information, Model S2):

(2) $$M_{ZFC}\left(T\right)=\;\overset{V_{c}\left(K_{eff},T\right)}{\underset{0}{\oint }}M_{S}L\left(\frac{MVH}{k_{B}T}\right)f\left(V\right)dV+\;\overset{\infty }{\underset{V_{c}\left(K_{eff},T\right)}{\oint }}M_{S}\frac{MH}{3K}f\left(V\right)dV$$

where *L*(*x*) is the Langevin function, *M* the domain magnetization, *Ms* the saturation magnetization, and *f*(*V*) is the size distribution. *V_c_* (*K_eff_*, *T*) represents the critical volume fraction of thermally-activated nanoparticles at a certain temperature. From the curve fittings (Figure 5), *K_eff_* at 0 K and mean diameters (*d_ZFC_*) with standard deviation have been obtained (Table 2).

The good agreements shown by the fittings corroborate the validity of the employed model where no contributions of dipolar interactions have been taking into account. The samples Co_0.15__60, Co_0.15__90, and Co_0.15__105 with the maximum mean <*T_B_*> values, which are related to bigger nanoparticles, also present the highest values of anisotropy constants (138, 55, and 208 kJ/m^3^, respectively). This fact is explained because of the highest contents of Co^2+^ for these nanoparticles (*x* > 0.09). The Co^2+^ ion is expected to increase the anisotropy energy in the ferrite lattice, particularly when it occupies Oh sites characterized by the three-fold orbital degeneration, ^4^T_1g_ ground state, and a large spin-orbit coupling. This tendency is clearly observed for the Co_0.01__60, Co_0.04__60, Co_0.1__60, and Co_0.15__60 samples where the values of anisotropy energy constants, 46.5, 54.9, 102, and 138 kJ/m^3^, respectively, gradually increase with increasing cobalt content, as has been previously observed for similar phases [14]. Nevertheless, the introduction of Co^2+^ affects to all the synthesized samples as the anisotropy values obtained are higher than those observed for nanosized magnetite [38], but lower than anisotropy constants obtained for CoFe_2_O_4_ nanoparticles [11].

As in cobalt-doped samples, the contribution of magneto-crystalline anisotropy can be important and expected to be cubic, it is assumed that *K_eff_* = *K_c_*/4 [39,40,41,42]. The values obtained vary in the range between 428 and 835 kJ/m^3^, which have been used to calculate the theoretical content of cobalt in these ferrites (0.02 < *x* < 0.08), with the formula proposed by Zhang et al. [34] (Table 2). Although smaller values than those obtained from ICP appear, it is notable that these values would reflect estimated Co contents in a magnetite unit cell and ICP values of the overall concentrations, in spite of being inside the cell or as a segregated phase. For samples with a theoretical content of Co *x* = 0.15, the cobalt proportion obtained from ZFC-FC curves maintains the value around 0.045, regardless the time of reflux. In the case of Co*_x_*_60 samples, the diminution of Co from 0.055 to 0.02 is in good accord with the general trend of theoretical decreasing amounts of Co in the samples (*x* = 0.15, 0.1, 0.04 and 0.01).

**Figure 6:**
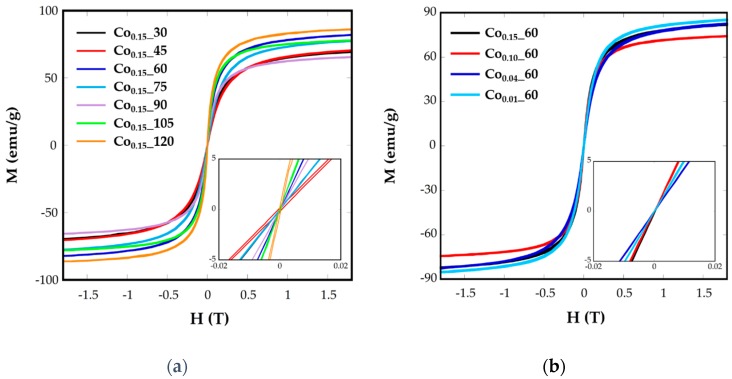
Room-temperature hysteresis loops for samples Co*_x_*_*t* at different (**a**) reflux times and (**b**) Co concentrations.

The superparamagnetic behavior of ferrite nanoparticles was confirmed by the absence of the coercive field (*H_c_*) and remanence magnetization (*M_r_*) at room temperature, as can be observed in the plots of magnetization, *M*, vs. the applied field, *H*, at 250 K (Figure 6). It can be observed that magnetization does not reach saturation in any case, even at the highest applied field. As the magnetic interaction can be explained by the superexchange interaction between the tetrahedral (A) and octahedral (B) sites, the magnetic moment per formula *M* would be the difference between the magnetic moments of both sites, *M* = *M_B_* – *M_A_*. In this sense, the substitution of Fe^2+^(4 μ_B_) by Co^2+^(3 μ_B_) in the partially-inverse structure, with higher preference of Co^2+^ for B sites [11], would induce a decrease in net magnetization, as can be observed for all the samples. The saturation magnetization values at R.T. for Co*_x_*_*t* samples vary from 86 to 65 emu/g (Table 2), lower than magnetite bulk materials (92 emu/g) and lower than pure and crystalline magnetite nanoparticles [6].

The different values from one sample to another could be related to the different cobalt substitution in the samples, as has been deduced from ICP analysis, or to the spin canting due to sub-coordinated surface atoms [15].

Hysteresis loops have also been recorded at 5 K (Figure S4), where coercive values (*H_c_*) have been calculated (Table 2). These values vary from 610 to 6360 Oe for the nanoparticles Co*_x_*_*t* and it is expected to change with the microstructural differences between the samples. Increasing *H_c_* values, up to 1130 Oe, have been observed for Co stoichiometries in the range of 0.1 < *x* < 1 [39]. In our case, Co_0.15__60 and Co_0.15__105 samples with the highest nominal cobalt contents (0.14 and 0.16, respectively) present the highest *H_c_* values, 5300 and 6360 Oe, respectively. In the other samples, together with the influence of cobalt content another effects as the grain size and different morphology could also affect coercive fields.

### 2.4. Electron Magnetic Resonance

**Figure 7:**
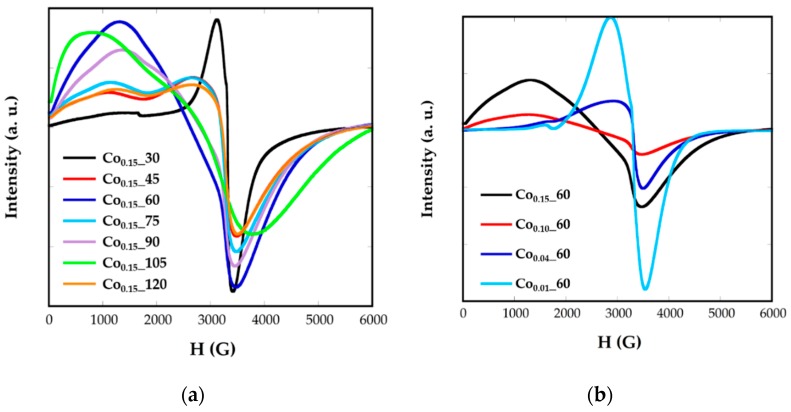
EMR spectra for synthetized samples Co*_x_*_*t* at different (**a**) reflux times and (**b**) Co concentrations.

Electron magnetic resonance (EMR) measurements were carried out to obtain complementary information for the magnetic behavior. Figure 7 shows the EMR spectra recorded for the Co*_x_*_*t* samples in toluene colloidal dispersions. Samples of Fe_3_O_4_ nanoparticles, with homogeneous size, shape, and composition, are characterized by unique and well-resolved lines with a *g* factor = 2, when nanoparticles are small. The *g* factor is related with the magnetic field occurring at the maximum resonance, *H_r_*, by the following equation:

(3) $$g=\frac{h\nu }{\beta H_{r}}$$

where *h* is Planck’s constant, ν is the microwave frequency, and *β* is the Bohr magneton. It has been previously observed that both the broadness of the line and the value of the resonant line, *H_r_*, varies for magnetite NPs from one sample to another and that this variation is strongly correlated with nanoparticle size [6].

Nevertheless, in Co*_x_*_*t* nanoparticles, broad and asymmetric signals not centered at *g* = 2 are obtained, presenting slight variations from one sample to another as the cobalt contents, the size, or morphology change. This fact would corroborate the presence of cobalt-doped ferrite nanoparticles. In all the spectra two components appear, one at high resonant fields and another one at lower ones. This asymmetry in the signals could be related with the presence of cobalt, with high anisotropy energy, which could induce the orientation of the nanoparticles to the magnetic field in particular directions. In this sense, the values of *g* corresponding to the maximum resonance magnetic field, *H_r_* is in the range *g* = 1.78–1.94 and the *g* factors corresponding to the minimum resonance magnetic field, *H_r_*, are between 4.96 and 8.28. It can be noticed that samples with similar signals also have close shape, size, and cobalt content, as Co_0.15__45, Co_0.15__75, and Co_0.15__120 present sizes of 6 nm and cobalt contents calculated from *ZFC*-*FC* measurements of *x* = 0.04 (Table 2).

## 3. Materials and Methods

### 3.1. Materials

Iron(III) acetylacetonante (Fe(acac)_3_, 99.0%), cobalt(II) acetylacetonate (Co(acac)_2_, 97%), oleic acid (OA, 90%), oleylamine (OLA, 87%), benzyl ether (98%), 1,2 hexadecanediol (HDD, X%) and toluene (X%) were purchased from Sigma-Aldrich (Madrid, Spain). Ethanol was purchased from Panreac (Barcelona, Spain). All the chemicals were used as received without further purification.

### 3.2. Syntheiss of Cobalt-Doped Nanoparticles

Cobalt-doped nanoparticles with different morphologies were prepared by thermal decomposition of Fe(acac)_3_ and Co(acac)_2_ in highly boiling organic solvent in the presence of oleic acid (OA), oleilamine (OLA), and 1,2-hexadecanediol (HDD). In detail, 25 mL of benzyl ether, 1.9 mmol Fe(acac)_3_, 0.1 mmol Co(acac)_2_, 4 mmol of OA and OLA, and 8 mmol of HDD were placed into a 250 mL three-neck flask. Other metal amounts were used as samples with cobalt content from 1% to 5%. The reaction mixture was mechanically stirred and de-gassed at room temperature at least for 10 min to remove the oxygen and water. The mixture was first heated at 200 °C for 30 min (nucleation phase) and then brought to reflux. The reflux time under an argon atmosphere (0.5–2 h) was varied in order to study the change of the magnetic properties and to relate them to their physicochemical characteristics. After heating, the reaction mixture turned black. Finished the growth phase, the solution was cooled to room temperature. The resulting nanoparticles were precipitated with ethanol, magnetically separated using a permanent Nd magnet and redispersed in 5 mL toluene. This procedure was repeated twice in order to remove all the benzyl ether from the nanoparticles. Finally, undesirable ferrite aggregates were removed by centrifugation at 4000 rpm over 80 min.

Iron and cobalt contents of samples were determined by inductively-coupled plasma-atomic emission spectroscopy (IPC-AES), using an ELAN9000 ICP-MS (Waltham, MA, USA) spectrophotometer. X-ray diffraction (XRD) of powder samples was made using a PANalytical X’Pert PRO diffractometer (Egham, Surrey, UK) equipped with a copper anode (operated at 40 kV and 40 mA), diffracted beam monochromator, and PIXcel detector. Scans were collected in the 5–70° 2θ range, with step size of 0.026° 2θ and 60 s per step. Thermogravimetric measurements were performed in a NETZSCH STA 449 C (Selb, Baviera, Germany) thermogravimetric analyzer by heating 10 mg of sample at 10 °C/min under a dry Ar atmosphere. TEM micrographs were obtained using a Philips CM200 microscope (Amstelplein, Amsterdam, The Netherlands) at an acceleration voltage of 200 kV. For preparing the samples, MNPs dispersed in toluene were drop-cast onto copper grids. EMR spectra were recorded on a Bruker ELESYS spectrometer (Silberstreifen, Rheinstetten, Germany) equipped with a standard Oxford low-temperature device operating in the X band. Magnetization measurements as a function of temperature after cooling at zero field and field (10 Oe, *ZFC*-*FC* curve) were performed in a commercial Quantum Design MPMS-5 SQUID (Darmstadt, Hesse, Germany) magnetometer. Hysteresis loops at room temperature were acquired using a homemade VSM magnetometer up to a maximum field of 18 kOe with high low-field resolution. Hysteresis loops at 5 K were performed in a VSM magnetometer from Cryogenic Ltd. (London, UK) up to a maximum field of 100 kOe.

## 4. Conclusions

This work provides a deeper insight into the obtaining of cobalt-doped ferrite nanoparticles with the Fe_3−*x*_Co*_x_*O_4_ (0 < *x* < 0.15) general formula. The ability to tune the amount of cobalt introduced in the ferrite lattice is demonstrated by adjusting the reflux time from 30 to 120 min with nanoparticles of a theoretical composition of Fe_2.85_Co_0.15_O_4_. This change in the preparation process seems not to significantly affect the average particle size or the particle size distribution, but influences the real cobalt content in the samples, as in all cases smaller amounts of cobalt have been confirmed by ICP analysis. Magnetic data confirm the dependence of the blocking temperature, *T_B_*, with the cobalt content. Thus, the higher magnetization is found with the longer reflux time and, consequently, when a higher amount of cobalt is incorporated in the ferrite nanoparticles. In order to prove the effect of cobalt content on the magnetic response another group of samples have been prepared changing the Fe/Co precursor ratio of Fe_3−*x*_Co*_x_*O_4_ (0 < *x* < 0.15) with the same reflux time of 60 min. In this sense, higher Co contents give rise to an increase in the coercive field at 5 K. Regarding the blocking temperatures, a similar trend has been observed. From fittings of ZFC branch curves, anisotropy energy constants and nominal contents of cobalt in the samples have also been obtained. Finally, EMR measurements have also confirmed cobalt-doped ferrites by broad and asymmetric signals not centered at *g* = 2.

## Figures and Tables

**Table 1:** Cell parameter, a (Å), obtained from the fitting of XRD patterns. Cobalt content in ferrites, determined by chemical analysis, percentage of surface coating calculated from thermal analysis, and particle sizes obtained from TEM analysis.

Sample	a (Å)	ICP	TGA (%)	TEM (nm)
Co_0.15__30	8.3757 (4)	Co_0.08_Fe_2.92_O_4_	28.08	6 (1)
Co_0.15__45	8.3701 (3)	Co_0.08_Fe_2.92_O_4_	30.68	6 (1)
Co_0.15__60	8.3780 (3)	Co_0.14_Fe_2.86_O_4_	43.82	8 (1)
Co_0.10__60	8.3769 (2)	Co_0.07_Fe_2.93_O_4_	29.58	7 (1)
Co_0.04__60	8.3700 (3)	Co_0.03_Fe_2.97_O_4_	23.31	7 (1)
Co_0.01__60	8.3730 (2)	Co_0.01_Fe_2.99_O_4_	25.77	6 (1)
Co_0.15__75	8.3671 (3)	Co_0.1_Fe_2.9_O_4_	40.85	7 (1)
Co_0.15__90	8.4277(4)	Co_0.09_Fe_2.11_O_4_	31.33	11 (1)
Co_0.15__105	8.3798 (2)	Co_0.11_Fe_2.89_O_4_	25.89	8 (1)
Co_0.15__120	8.3671 (5)	Co_0.16_Fe_2.84_O_4_	36.981	6 (1)

**Table 2:** Formula obtained from ICP calculations, average blocking temperature (<*T_B_*>), apparent magnetic diameter (*D_ZFC/FC_*), anisotropy constant *K_eff_*, (0 K) calculated from Equation (2), magneto-crystalline anisotropy *K_c_*, calculated cobalt content (Co*_x_*) from *K_c_* values, coercive field (*H_c_*) and magnetization saturation at 250 K (*M_s_*) for Co*_x_*_*t* synthetized samples.

Sample	ICP	<*T_b_*> (K)	*D_ZFC_*_/*FC*_ (nm)	*K_eff_* (0 K) (KJ/m^3^)	*K_c_* (KJ/m^3^)	Co*_x_*	*H_c_* (5 K) (Oe)	*M_s_* (emu/g)
Co_0.15__60	Co_0.14_Fe_2.86_O_4_	75.7	7.51 (1)	138	553.2	0.05	5.300	82.15
Co_0.1__60	Co_0.07_Fe_2.93_O_4_	66.7	7.68 (1)	102	434	0.04	3650	74.25
Co_0.04__60	Co_0.03_Fe_2.97_O_4_	34.8	7.39(1)	54.9	219.6	0.02	999	82.59
Co_0.01__60	Co_0.01_Fe_2.99_O_4_	24.6	6.86 (1)	46.5	186.2	0.02	610	85.25
Co_0.15__30	Co_0.08_Fe_2.92_O_4_	51.8	6.37 (2)	115.6	462.4	0.05	3.300	69.66
Co_0.15__45	Co_0.08_Fe_2.92_O_4_	47.8	6.14 (1)	107	428	0.04	3.600	70.58
Co_0.15__75	Co_0.1_Fe_2.9_O_4_	46.1	6.44 (1)	102.4	409.6	0.04	3.310	77.48
Co_0.15__90	Co_0.09_Fe_2.11_O_4_	96.5	10.7 (2)	55.1	220.6	0.02	2.262	65.63
Co_0.15__105	Co_0.11_Fe_2.89_O_4_	110.2	7.96 (1)	208.8	835.2	0.08	6.360	77.93
Co_0.15__120	Co_0.16_Fe_2.84_O_4_	49.9	5.98 (2)	120.9	483.6	0.05	1.800	86.16

## Supplementary Materials

The following are available online at http://www.mdpi.com/2079-4991/8/2/63/s1, Model S1. Effective anisotropy constant, *K_eff_*, calculation within the non-interacting super-paramagnetic (SPM) model. Model S2. Determination of Anisotropy Constant. Figure S1. X-ray diffraction pattern of the residue of the Co_0.15__60 sample obtained at TGA. Figure S2. Particle size distributions of Co_0.15__30, Co_0.15__45, Co_0.15__60, Co_0.10__60, Co_0.04__60, Co_0.01__60, Co_0.15__75, Co_0.15__90, Co_0.15__105, and Co_0.15__120. Figure S3. Magnetic susceptibility (*ZFC* and *FC*) measured at 10 Oe and derivative −*d*(χ*_FC_*-χ*_ZFC_*)/*dT* of (a) Co_0.15__30, (b) Co_0.15__45, (c) Co_0.15__60, (d) Co_0.15__75, (e) Co_0.15__90, (f) Co_0.15__10, (g) Co_0.15__120, (h) Co_0.10__60, (i) Co_0.04__60, and (j) Co_0.01__60. Figure S4. Hysteresis loops at 5 K for the samples obtained with different reflux times (left) and Co contents (right).
