# Supplementary material for: Exploring Reaction Conditions to Improve the Magnetic Response of Cobalt-Doped Ferrite Nanoparticles
Source: Nanomaterials (Basel). 2018 Jan 25;8(2):63. doi: 10.3390/nano8020063 (PMC5853696; doi:10.3390/nano8020063)
Supplement: Supplementary file 1 [file nanomaterials-08-00063-s001.pdf]

nanomaterials

MDPI

*Supplementary Information*

# Exploring Reaction Conditions to Improve the Magnetic Response of Cobalt-Doped Ferrite Nanoparticles.

**Itziar Galarreta [1], Maite Insausti [1,2,*], Izaskun Gil de Muro [1,2], Idoia Ruiz de Larramendi [1] and Luis Lezama [1,2]**

[1] Dpto. de Química Inorgánica, Universidad Del País Vasco, UPV/EHU, E-48080 Bilbao, Spain
[2] BCMaterials, Parque Científico y Tecnológico de Bizkaia, E-48160 Derio, Spain
* Correspondence: maite.insausti@ehu.eus; Tel.: +34-94-601-2703

## SUPORTING INFORMATION

***Model S1***. Effective anisotropy constant, Keff, calculation within the Non-Interacting Super-Paramagnetic (SPM) model.

**Model S2.** Determination of Anisotropy Constant.

**Figure S1.** X-Ray diffraction pattern of the residue of the $Co_{0.15}$_60 sample obtained after the thermogravimetric analysis

**Figure S2.** Particle size distributions of Co0.15_30, Co0.15_45, Co0.15_60, Co0.10_60, Co0.04_60, Co0.01_60, Co0.15_75, Co0.15_90, Co0.15_105 and Co0.15_120.

**Figure S3.** Magnetic susceptibility (ZFC and FC) measured at 10 Oe and derivative $-d(\chi_{FC}-\chi_{ZFC})/dT$ of (a) $Co_{0.15}$_30, (b)$Co_{0.15}$_45, (c)$Co_{0.15}$_60, (d)$Co_{0.15}$_75,(e)$Co_{0.15}$_90, (f)$Co_{0.15}$_10, (g)$Co_{0.15}$_120., (h)$Co_{0.10}$_60, (i)$Co_{0.04}$_60 and (j)$Co_{0.01}$_60.

**Figure S4**. Hysteresis loops at 5 K for the samples obtained with different reflux times (left) and Co contents (right).

**Model S1. Effective anisotropy constant, Keff, calculation within the Non-Interacting Super-Paramagnetic (SPM) model.**

In a set of uniaxial magnetic single domains of size D oriented at random, neglecting the dipolar interaction, the effective anisotropy constant is proportional to the so-called blocking temperature ($T_B$):

$$K_{eff} = \frac{k_B \ln(\tau_m / \tau_0)}{V} T_B \quad (1)$$

TB becomes a direct experimental measurement of the energy barrier between the two ground states "up" and "down" of the particle magnetic moment (KV). In equation (1), τm is the characteristic time of the experiment (time window) and τ0 is the inverse of the natural fluctuation rate of the particle magnetic moment.

In a measurement of DC magnetization ln(τm /τ0) ≈ 25 , so it follows that, assuming a set of particles of identical size, the effective anisotropy constant can be directly deduced from TB as:

$$K_{eff} = \frac{25 k_B}{V} T_B \quad (2)$$

In such an ideal system, TB coincides exactly with the maximum of the ZFC curve. When the natural dispersion of sizes is taking into account, equation (2) turns into the following one:

$$K_{eff} = \frac{25 k_B}{V} \langle T_B \rangle \quad (3)$$

where ⟨ $T_B$ ⟩ is the average of the blocking temperatures of the population, each one depending on the size of a given particle. It is to note that ⟨ $T_B$ ⟩ does not lie at the maximum of the ZFC, in a set of particles with some dispersity.

In order to calculate the average blocking temperature, determination of the $f(T_B)$ (proportional to the energy barrier distribution) is necessary. It can be obtained experimentally from the ZFC/FC measurement of magnetization under a sufficiently small-applied field, considering that:

$$f(T_B) \approx \frac{d}{dT}(M_{FC} - M_{ZFC}) \quad (4)$$

In this way and after normalizing the derivative of the difference between ZFC and FC with the condition: ∫f($T_B$)d $T_B$ =1, the average blocking temperature is given by:

$$\rangle T_B \langle = \int_0^{\infty} T f(T_B) dT_B \quad (5)$$

**Model S2.** Determination of Anisotropy Constant.

**Fit of ZFC/FC measurements**

A simple non-interacting model has been used for the fit, in which the population of MNPs (given by a size distribution $f(D)$) is sharply divided in two groups at each temperature, depending on their particular size: the fraction in an ideal superparamagnetic state that corresponds to MNPs below a certain critical volume and those, above such limit, whose super spin remains blocked:

$$M_{ZFC}(T) = \int_0^{v_c} M_S L(\frac{MV\mu_0 H}{k_B T}) f(D) \mathrm{d}V + M_S \frac{M\mu_0 H}{3K_{U,C}} f(V) \mathrm{d}V \quad (6)$$

In the first term, we make use of the low energy barrier approximation where the energy barrier (defined as $K_{eff} V$, being $V$ the particle volume) is much smaller than the thermal energy ($k_B T$ where $k_B$ is the Boltzmann Constant) and so can be omitted. Accordingly, the response of the magnetization to changes of magnetic field or temperature ($H$ or $T$) follows a Langevin function, where $M$ is the particle magnetization (A/m in S.I.) and $M_S$ is the experimental saturation magnetization (including non-magnetic mass contribution, in general). Both the experimental magnetization and the particle magnetization are allowed to decrease with temperature following a spin wave-like behavior (Bloch type law) as:

$$M(T) = M(0)\mathrm{e}^{-BT3/2} \quad (7)$$

where the so-called Bloch constant (B) has been obtained from the magnetization measurements as a function of temperature under the maximum field of 7T, being between 2 and $4 \times 10^{-5}$ in all cases.

The second term component results from the initial susceptibility of a randomly oriented magnetic domains either with uniaxial ($K_U$) or with cubic anisotropy ($K_C$) provided that $K_C > 0$. Note that $K_C$, is the first cubic anisotropy and is equal to $4K_{eff}$ if $K_C > 0$ as in Co ferrite. The threshold between the two populations (it is limiting both integrals) is given by a critical diameter or volume ($D_C/D_v$) such that:

$$V_C(T) = \frac{25 k_B T}{K_{eff}(T)} \quad (8)$$

In this model, the position and shape of the ZFC maximum depends on the anisotropy through this critical volume that depends explicitly on temperature and also implicitly, through the function $K_{eff}(T)$ which is given by different models as stated in the manuscript, depending on the relative content of Co ferrite.

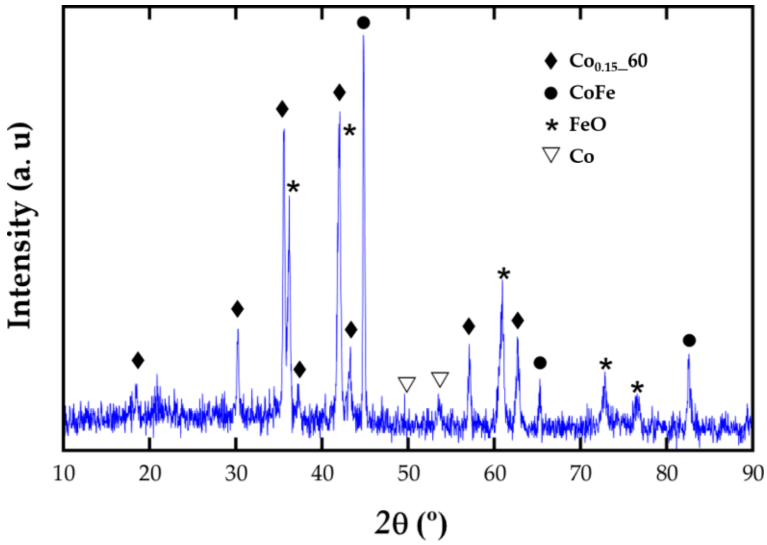


**Figure S1.** X-Ray diffraction pattern of the residue of the $Co_{0.15}$_60 sample obtained after the thermogravimetric analysis

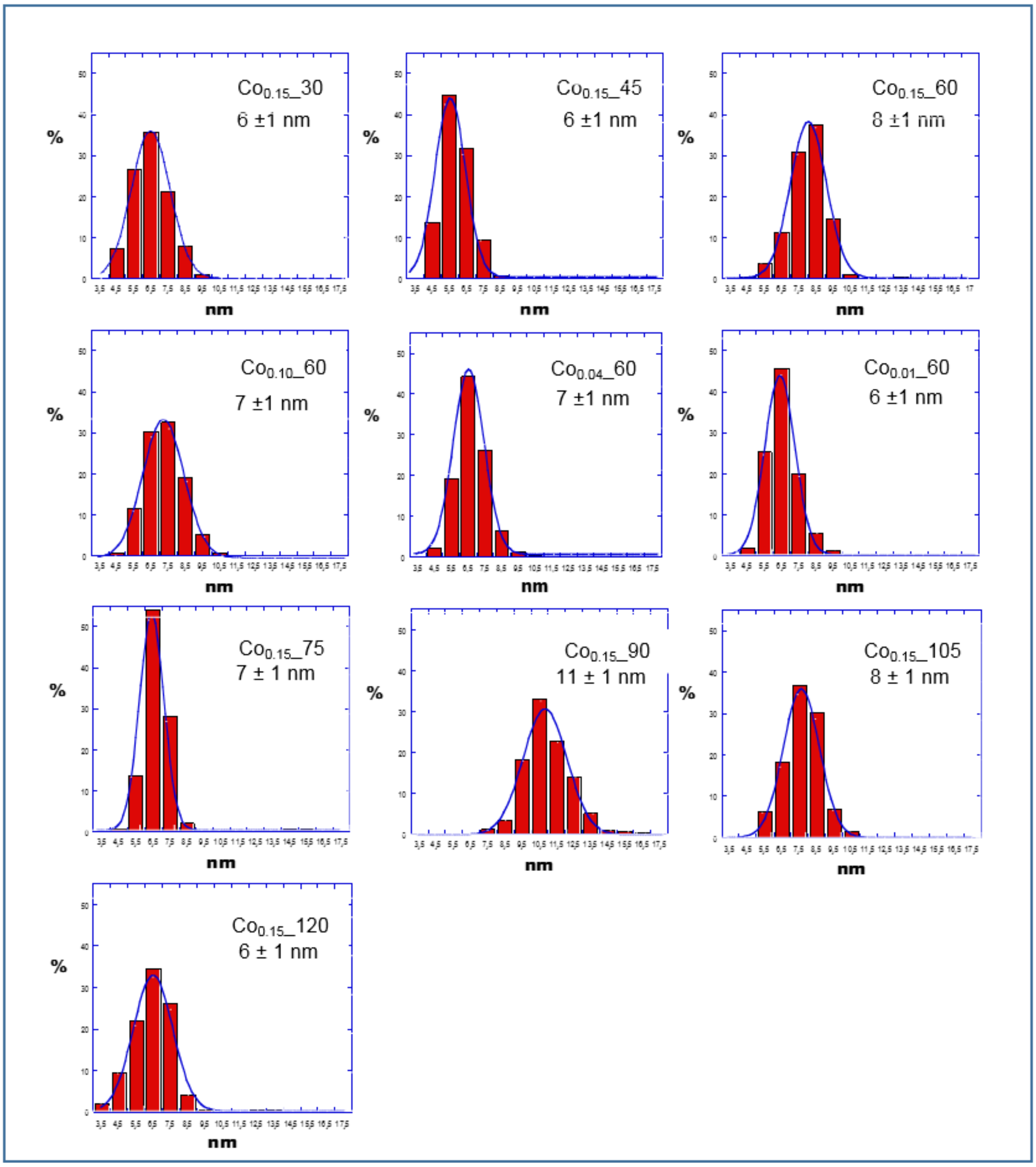


**Figure S2.** Particle size distributions of Co$_{0.15}$_30, Co$_{0.15}$_45, Co$_{0.15}$_60, Co$_{0.10}$_60, Co$_{0.04}$_60, Co$_{0.01}$_60, Co$_{0.15}$_75, Co$_{0.15}$_90, Co$_{0.15}$_105 and Co$_{0.15}$_120.

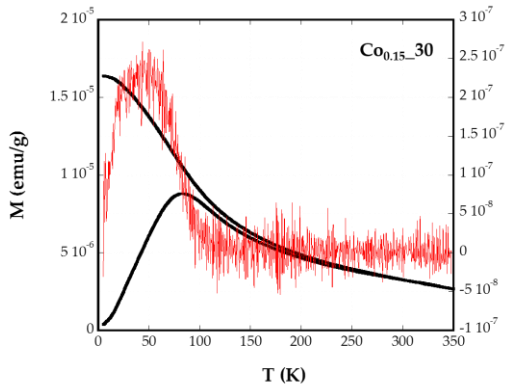


**(a)** Co0.15_30

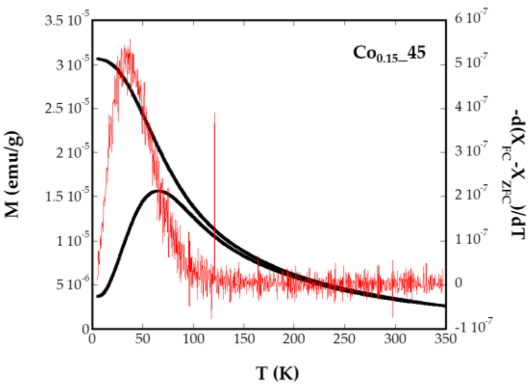


**(b)** Co0.15_45

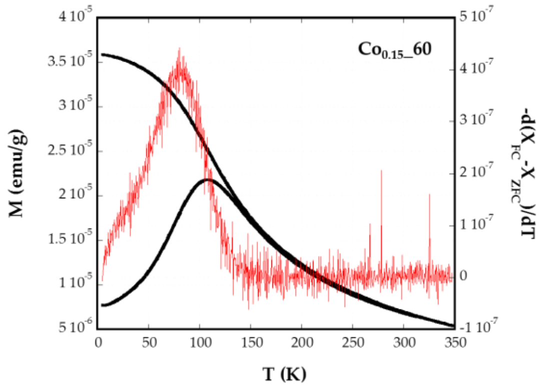


**(c)** Co0.15_60

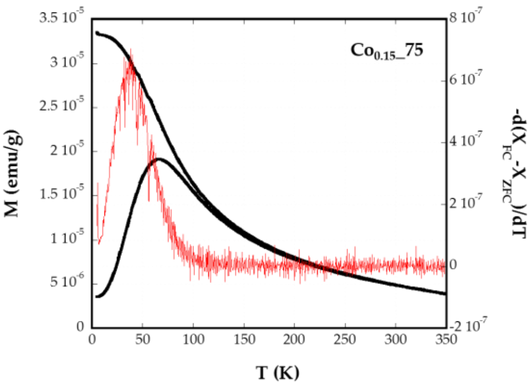


**(d)** Co0.15_75

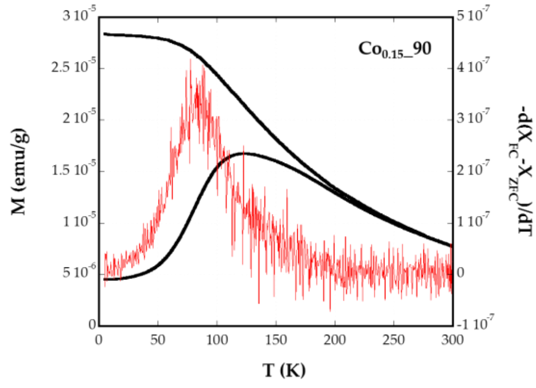


**(e)** Co0.15_90

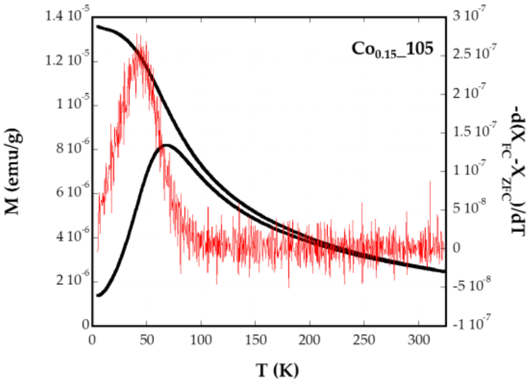


**(f)** Co0.15_105

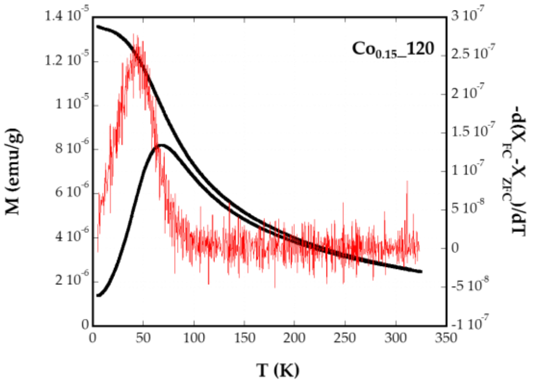


**(g)** Co0.15_120

**Figure S3.** Magnetic susceptibility (ZFC and FC) measured at 10 Oe and derivative –d($\chi_{FC}$-$\chi_{ZFC}$)/dT of (a) $Co_{0.15}$_30, (b) $Co_{0.15}$_45, (c) $Co_{0.15}$_60, (d) $Co_{0.15}$_75,(e) $Co_{0.15}$_90, (f) $Co_{0.15}$_105 and (g) $Co_{0.15}$_120.

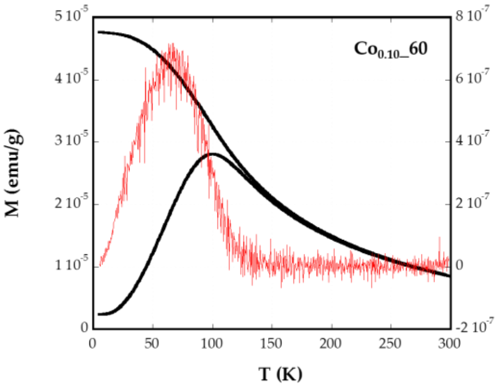


**(a)** $Co_{0.10}$_60

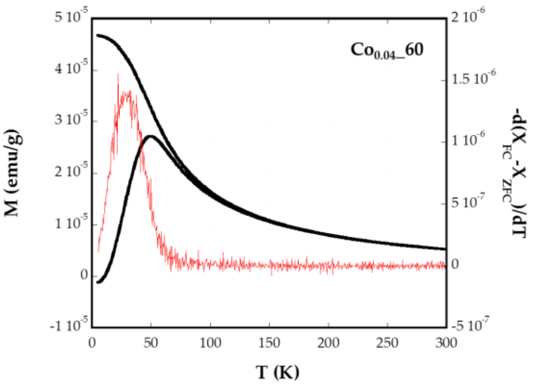


**(b)** $Co_{0.04}$_60

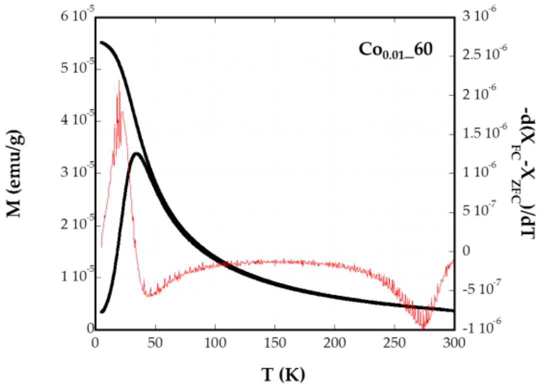


**(c)** $Co_{0.01}$_60

**Figure S3 (continued).** Magnetic susceptibility (ZFC and FC) measured at 10 Oe and derivative –d($\chi_{FC}$-$\chi_{ZFC}$)/dT of (a) $Co_{0.10}$_60, (b) $Co_{0.04}$_60 and (c) $Co_{0.01}$_60.

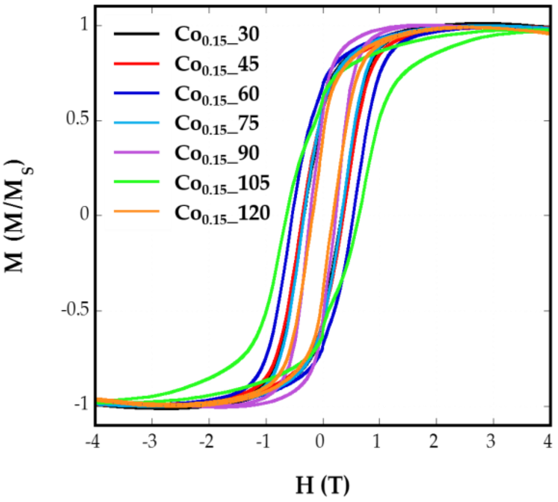


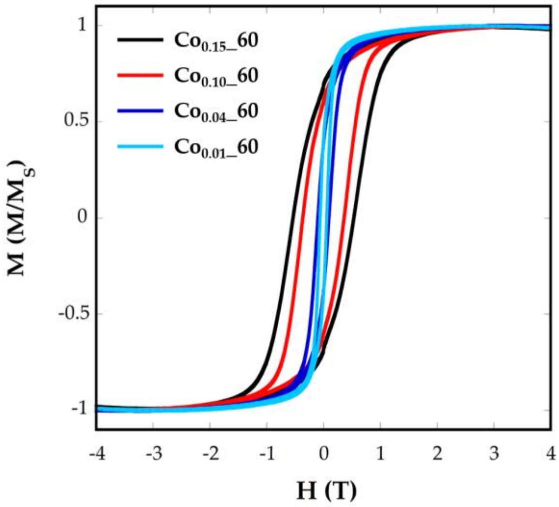


**Figure S4**. Hysteresis loops at 5 K for the samples obtained with different reflux times (left) and Co contents (right).

© 2018 by the authors. Submitted for possible open access publication under the terms and conditions of the Creative Commons Attribution (CC BY) license (http://creativecommons.org/licenses/by/4.0/).
